# Long-term outcome in 324 polytrauma patients: what factors are associated with posttraumatic stress disorder and depressive disorder symptoms?

**DOI:** 10.1186/s40001-017-0282-9

**Published:** 2017-10-30

**Authors:** Lisa Falkenberg, Christian Zeckey, Philipp Mommsen, Marcel Winkelmann, Boris A. Zelle, Martin Panzica, Hans-Christoph Pape, Christian Krettek, Christian Probst

**Affiliations:** 1 Trauma Department, Hannover Medical School, Hannover, Germany; 2 Department of General, Trauma and Reconstructive Surgery, Ludwig-Maximilians-Universität München, Munich, Germany; 3 Division of Orthopaedic Traumatology, Department of Orthopaedic Surgery, University of Texas Health Science Center at San Antonio, San Antonio, TX USA; 4 Department of Trauma Surgery, University Hospital Zurich, Zurich, Switzerland; 5 Department of Trauma and Orthopaedic Surgery, Cologne-Merheim Medical Center, University of Witten/Herdecke, Cologne, Germany

## Abstract

**Background:**

Physical impairment is well-known to last for many years after a severe injury, and there is a high impact on the quality of the survivor’s life. The purpose of this study was to examine if this is also true for psychological impairment with symptoms of posttraumatic stress disorder or depression after polytrauma.

**Design:**

Retrospective cohort outcome study.

**Setting:**

Level I trauma centre.

**Population:**

637 polytrauma trauma patients who were treated at our Level I trauma centre between 1973 and 1990. Minimum follow-up was 10 years after the injury.

**Methods:**

Patients were asked to fill in a questionnaire, including parts of the Posttraumatic Stress Diagnostic Scale, the Impact of Event Scale-Revised and the German Hospital Anxiety and Depression Scale, to evaluate mental health. Clinical outcome was assessed before by standardised scores.

**Results:**

Three hundred and twenty-four questionnaires were evaluated. One hundred and forty-nine (45.9%) patients presented with symptoms of mental impairment. Quality of life was significantly higher in the mentally healthy group, while the impaired group achieved a lower rehabilitation status.

**Conclusions:**

Mental impairment can be found in multiple trauma victims, even after 10 years or more. Treating physicians should not only focus on early physical rehabilitation but also focus on early mental rehabilitation to prevent long-term problems in both physical and mental disability.

## Background

In the last decades, the survival rate of multiple trauma patients has increased to 85–88%. This is due to progress in prehospital treatment with shortened rescue times, optimised intensive care treatment, established special trauma centres, and improved surgical care [1–4].

Investigation of long-term outcomes after multiple trauma injuries is not only important for quality control but also important for economic interest, as a substantial amount of lost productivity comes from within the full-time working population. The average age of polytraumatised patients ranges from 20 to 60 years. The male-to-female ratio is approximately 3–1 and men frequently sustain more severe injuries [5–7].

Previous studies with a follow-up of 12–28 months found female gender [8–10] and lower age [11, 12] to be associated with the development of mental impairment after severe physical trauma. Economically, in addition to costs for the treatment of the patients, a financial burden results from the loss of working time caused by death, working disability, successive costs of rehabilitation or disability caused by mental impairment. A good functional status after discharge is related to a higher return to work rate [13]. This is not only referred to as physical impairment but also as psychological impairment to a considerable degree [14].

Even though the above information reflects good understanding of the problem of mental impairment after physical trauma up to 8 years of follow-up, the role of time including further therapy during the initial years after injury, the adoption to changes over several years and the very long-term course remains unclear. Therefore, our study is conducted to evaluate the incidence and to detect risk factors for the development of the two most common psychiatric sequelae of trauma—posttraumatic stress disorder (PTSD) and major depressive disorder (MDD) more than 10 years after major trauma in adults.

## Patients and methods

### Participants

All patients included were recruited from the “Hannover Rehab Study” [15]. Study design proceeded in two stages. In the first stage, patients treated at our Level I trauma centre between 1973 and 1990 were recruited to assess their QoL and physical rehabilitation status. The assessment and re-examination of patients was performed from January 2000 to February 2003. The Institutional Review Board and the Ethics Committee of Hannover Medical School approved the study Protocol No. 2325-2000/03/22. All of the participants provided written informed consent. Inclusion criteria for the sample selection were as follows [15, 16]:

- Patients with multiple blunt injuries, or “polytrauma”.
- Patients treated at our Level I trauma centre between 1973 and 1990.
- Age 3–60 years at the time of injury.
- No other major injury/major negative life event (e.g. death of kin or divorce) until follow-up.

A polytrauma was considered when the following criteria were fulfilled: injuries to at least two long bones, one life-threatening injury with at least one additional injury and severe head trauma with at least one additional injury [17].

Six hundred and thirty-seven patients (67.8% of the potential enrollees; 75% male) attended the highly standardised interview by questionnaire and similarly standardised physical examination (BAZ, MP) on an out-patient basis. The reasons why the other patients were not included were administrative problems, medical problems or lack of interest in study participation. Details on this study were published earlier [15, 18].

The second stage of this study was performed as a follow-up in 2004 and evaluated the psychological outcome by questionnaire. All of the 637 patients were contacted by a letter and were asked to complete a self-report questionnaire. With an additional reminding letter, we finally received a response of 337 questionnaires (53%) that could be evaluated for psychological sequelae of polytrauma. Considering a possible lack of awareness, we excluded 13 patients who were younger than 10 years old at the time of trauma. The results presented in this paper are based on the data of the resulting 324 patients. In a comparison between participants and refusers, we found no difference in gender, age, injury severity or pattern, rehab treatment or physical outcome scores.

### Measures

Injury-related variables were assessed using the following instruments:

- The severity of injury was categorised by the Injury Severity Score (ISS) and New Injury Severity Score (NISS) (range 0–75), which were calculated based on the Abbreviated Injury Scale (AIS, score 1–6) [19, 20].
- The Glasgow Outcome Scale (GOS) [21] was used to assess the outcome after severe brain damage with a range of 1 (= death) to 5 (= good recovery).

The evaluation of the physical outcome included a personal interrogation by questionnaire and a head-to-toe physical examination by an orthopaedic surgeon. The measuring instruments used were the Short Form-12 (SF-12), a physical and psychological scale that is a modified version of the Short Form-36 (SF-36) in German [22, 23] to evaluate the health-related QoL (the “subjective satisfaction” with the patient’s own overall rehabilitation status) [15], and the Hannover Score for Polytrauma Outcome (HASPOC). The latter instrument was developed to specifically classify the status of rehabilitation of polytraumatised patients [24]. Briefly, the HASPOC consists of a self-assessment part that contains a detailed patient questionnaire on individual, social, leisure, financial, professional and medical items and a provider report that summarises the results of a physical exam.

The questionnaire of the succession study included parts of well-established instruments to detect mental health status. It consisted of the Posttraumatic Stress Diagnostic Scale (PDS) [25], the Impact of Event Scale-Revised (IES-R) [26] and the Hospital Anxiety and Depression Scale (HADS-D) [27]. The PDS assessment parallels the Diagnostic and Statistical Manual of Mental Disorders (DSM-IV) diagnostic criteria and is a self-reported measure of PTSD. The IES-R is also a self-reported measure that assesses subjective distress caused by traumatic events. It contains seven additional items related to the hyperarousal symptoms of PTSD, which were not included in the original IES. The HADS-D is the German version of the HADS. It is a self-assessment scale designed to detect the symptom severity of anxiety disorders and depression in both psychiatric/primary care patients and the general population [28].

### Statistical analysis

For assessment purposes, we subdivided our population into two groups: the “healthy” group with no signs of mental impairment (i.e. the succession study questionnaires were negative for any of the three entities) and the “impaired” group that scored positive on the PDS, HADS-D and/or IES-R questionnaires. Therefore, scoring positive on any of the scales was interpreted as “showing symptoms” of mental impairment (PTSD or MDD or both illnesses).

Statistical analyses were performed in close cooperation with the Institute for Biometrics at Hannover Medical School using SPSS 13.0 ^®^ (SPSS Inc., Chicago, IL, USA). Differences between groups were regarded significant at the 95% probability level (referred to as *p* < 0.05). Results were given as mean ± standard deviation unless otherwise specified.

## Results

Table 1 presents the demographic data of the study population

**Table 1:** Demographic data

	Healthy (*n* = 175)	Impaired (*n* = 149)	*p* value
Females (*n*)	43 (24.6%)	40 (26.9%)	0.73
Age	27.5 ± 11.4	30.0 ± 12.2	0.07
Follow-up time (years)	17.9 ± 4.9	17.0 ± 4.9	0.73

Table 2 displays the injury-related data. The overall injury severity does not differ significantly between the two groups. Even more, the rate of traumatic brain injury (TBI) is comparable between both groups. However, values of the GOS < 5 at discharge from the initial hospital stay are associated with mental impairment at follow-up.

**Table 2:** Trauma-related factors

	Healthy	Impaired	*p* value
ISS	21.9 ± 10.0	21.1 ± 9.3	0.43
NISS	26.8 ± 12.0	25.1 ± 10.4	0.17
Traumatic brain injury (*n*)	112 (64%)	91 (61.07%)	0.67
GOS < 5 (*n*)	8 (4.6%)	21 (14.1%)	0.008
Stay at ICU (days)	15.5 ± 14.4	15.6 ± 19.3	0.96
Stay at ward (days)	29.3 ± 23.5	31.0 ± 24.5	0.61
Patients with physical rehabilitation (*n*)	90 (51.4%)	99 (66.4%)	0.01

*GOS* Glasgow Outcome Scale, *ISS* Injury Severity Score, *NISS* New Injury Severity, *ICU* Intensive Care Unit

Table 3 illustrates the psychological and physical outcomes. Significant differences can be found regarding the results of both the SF-12 (mental and physical) and the HASPOC of patient, physician and total between our two groups. The subjective satisfaction of our patients with their rehabilitation is another parameter that was tested. There is a significantly higher percentage among the healthy probands who reported satisfaction with their rehabilitation (healthy: 88.6%; impaired: 66.4%; *p* < 0.0001).

**Table 3:** Physical and mental outcome

	Healthy	Impaired	*p* value
HASPOC-patient	38.4 ± 26.7	64.8 ± 29.5	< 0.0001
HASPOC-physician	12.4 ± 15.2	20.8 ± 19.0	< 0.0001
HASPOC-total	50.8 ± 38.9	85.7 ± 43.9	< 0.0001
SF-12-MCS	53.6 ± 7.4	47.5 ± 9.8	< 0.0001
SF-12-PCS	46.4 ± 9.5	39.8 ± 9.9	< 0.0001

*SF-12-MCS* Mental Component Summary Score, *SF-12-PCS* Physical Component Summary Score

Table 4 shows some socioeconomic and job-related items surveyed at follow-up to allow for more detailed information on the current status of the participants’ lives. Significantly higher numbers of impaired patients report losing partners, friends or finances after the trauma. In addition, significantly higher numbers of impaired patients report requiring psychiatric treatment, difficulty in working and dissatisfaction with their rehabilitation status. No significant differences between the groups can be found concerning the additional number of smokers through the accident or regarding weight increase. The same refers to change of occupation or professional re-training in both groups.

**Table 4:** Qualities extracted from the HASPOC examination

Status at follow-up	Healthy (*n*)	Impaired (*n*)	*p* value
Loss of friends	19 (10.8%)	50 (33.6%)	< 0.0001
Financial loss	64 (36.6%)	81 (54.6%)	0.002
Loss of partner	6 (3.4%)	16 (10.7%)	0.02
Psychiatric treatment	19 (10.8%)	38 (25.5%)	0.001
Inability to work	8 (4.6%)	18 (12.1%)	0.04
Factors tested but with no significant difference			
Weight increase	110 (56.8%)	98 (65.8%)	0.62
Started to smoke	34 (19.3%)	42 (28.2%)	0.08
Job change	127 (72.6%)	120 (80.5%)	0.12
Professional re-training	20 (11.4%)	28 (18.8%)	0.57

## Discussion

The aim of this study is to describe the psychological long-term effects of major physical trauma and to detect factors associated with mental impairment after 14 years of follow-up. Several authors report a high prevalence of concurrent PTSD and MDD [29] at 8 months and 4–6 years after trauma [30, 31]. There are different hypotheses about the relation between both affections. Bleich et al. assume a shared predisposition to PTSD and MDD with the traumatic experience serving as a trigger. They base this theory on the chronological relationship of both disorders and the reports of their patients. Other theories explain the link between PTSD and MDD with the development of MDD as a secondary consequence of PTSD [32]. Because of the symptom overlap and concomitance, a clear distinction between both disorders can be difficult. In consideration to this, we combined all patients with either a PTSD or a depressive disorder or both into the one group with symptoms. The “healthy” control group consists of individuals without these diagnoses. In the search for recent literature, we mostly find studies referring to either PTSD or depressive disorders with a definitely higher proportion of PTSD studies. As a result, a direct comparison to other studies is not always possible. Also, the time period of observation after physical trauma is much shorter in the majority of the studies. However, we apply literature with similar issues to place our findings into context.

Our main findings can be summarised as follows:

1. Polytraumatised patients still show symptoms of mental impairment more than 14 years after the initial physical trauma.
2. Gender, age or injury severity does not affect the psychological outcome in our population.
3. Only the injury-related variables “GOS < 5” and “need for physical rehabilitation” are associated with mental impairment.
4. Individuals without mental impairment achieve higher scores in overall rehabilitation and health-related QoL and show more satisfaction with their rehabilitation results.

Contrary to our results and recent literature, women are found to be more vulnerable for psychological impairment such as PTSD after trauma when compared to men [12, 33, 34]. When comparing different studies with regard to PTSD or depressive disorders, study populations and trauma types have to be considered as relevant and determining parameters [35, 36].

In general, men are more often confronted with accidents, non-sexual assault, combat and war, while women experience more assaultive violence in every day life [37]. As a result, researchers observe a greater risk for the development of PTSD in the female gender in the general US [38]. We assume that women in general tend to seek more psychological support compared to male. As a result, women have a better chance to recover from PTSD or MDD over the years. This may also be a further explanation for the similar gender distribution in our study groups.

As far as age, previous studies indicate opposite results. Soberg et al. described younger age as a predictor for PTSD symptoms in probands 2 years after severe multiple trauma. Others came to similar results in their studies about flood victims and victims of burn injuries [11, 39]. However, Zhang and Ho demonstrated that older age was a risk factor for PTSD in survivors of an earthquake after 2 months. An epidemiological study in Germany showed a higher prevalence of PTSD among older age groups, which is assumed to be a consequence of World War II (WWII) [40]. As results may vary from one trauma type and study population to another, a direct comparison between studies is not always possible.

As far as our study, the participants are involved into traffic accidents and non-assaultive events, which may be an explanation for different results compared to other studies. In the review of the available literature, researchers come to the conclusion that women are not at greater risk for the development of PTSD after a motor vehicle accident (MVA) compared to men [41].

Besides trauma type and study population, the observation period has to be taken into account. The long-term aspect of our study may be another reason for converse findings in comparison to short-term observation studies. Other study groups find that female gender is associated with PTSD after 6 months observation, but showed no difference after 1 year [10, 42]. Beck et al. confirm these results by finding no sex differences in the rate of chronic PTSD in MVA victims at 6 months or longer after the physical injury. The hypothesis is that there are different mediators of acute and chronic symptoms of PTSD. Factors such as the individual coping strategy and psychosocial resources may be responsible for either maintaining or recovering from chronic PTSD [12]. Soberg et al. prove that persons using avoidant coping strategies after severe multiple trauma are more vulnerable to PTSD. In contrast to other studies, we excluded patients under the age of 10 from the study population to avoid a possible influence through lack of awareness. We find that there were no significant differences of the mean age in both of our groups.

We find several studies examining the correlation between the severity of injury and the onset of PTSD. Our results are confirmed by several authors [34, 43–48] who found no positive correlation between the incidence of PTSD and a high ISS or NISS. In contrast, the study of Han et al. reported ISS as a strong predictor for the onset of postinjury depression in trauma survivors. Patients with an ISS ≥ 16 demonstrated twice as much depressive symptoms than patients with moderate injuries. Frommberger et al. reported similar findings—higher ISS scores in patients with psychiatric disorders compared to patients without psychiatric disorders following a MVA [47]. The differences in the results of the studies may be due to different methodologies. The studies vary in approaches for the assessment of diagnoses, recruitment of subjects or the observation period. For example, the study of Han et al. included only children and young adolescents aged 12–19 years, while the follow-up period of 6 months was very short in the study of Frommberger et al. [47].

Similar to our results, no significant differences in the appearance of PTSD between patients with or without a head injury have been found by other authors [49, 50]. Other researchers reported that head injury with following amnesia can be protective against the development of mental disorders [51, 52]. Similar results were obtained by other researchers after an extended period of unconsciousness in study probands [53]. They observed far more frequent intrusive memories in accident victims who had been conscious. This could also explain the results of Zatzick et al. investigated the influence of severe, moderate and mild TBIs on the prevalence of PTSD [54]. The findings state that more severe TBIs are associated with a diminished risk of PTSD. In contrast, Chossegros et al. provided a positive correlation between a head injury and subsequent PTSD [55]. Others find a link between posttraumatic amnesia and PTSD [56]. We do not have information on the presence of amnesia or the duration of unconsciousness accompanying the TBI in our population or the influence of long-term sedatives during the ICU stay. As a result, further investigations referring to this information are not possible in our study.

With regard to the GOS, the patients of the control group achieved significantly higher values and, therefore, less severe cerebral dysfunction. Levin et al. [57] reported similar results with more serious cognitive impairment in patients with MDD. A GOS of 3 correlates with severe disability, possibly with no improvement over the years that result in a higher risk for the development of a depressive disorder.

Our findings about the functional outcome and health-related QoL were as anticipated. The control group achieved higher values in the SF-12, which correlates with better health-related QoL. Regarding the HASPOC, higher values correspond with a poorer outcome (e.g. lower values with excellent overall rehabilitation outcome). Our results show a significantly poorer rehabilitation outcome among the mentally impaired probands, regarding both subjective and objective evaluations. Regardless of the method used, several studies observe functional impairments and decreases in health-related QoL in association with PTSD or MDD. A significant association exists between lower scores on the SF-36 physical component summary and the incidence of PTSD and MDD [58]. Others have reported unfavourable outcomes in QoL with correlations to PTSD, which demonstrates an association between partial PTSD and poor mental health functioning in veterans from Iraq and Afghanistan [59]. Also, other authors have seen decreases in health-related QoL in PTSD patients 2 years after injury [60]. Comparable to previous findings, the patient’s subjective opinion toward their rehabilitation result is statistically significant and more dissatisfied among patients in the “impaired” group.

Different conclusions can be drawn about the relationship between the overall rehabilitation outcome (objective and subjective) and potential psychopathological sequelae. One hypothesis is that an unsatisfactory rehabilitation status is a risk factor for the development of a mental disorder, because the patients get reminded daily of the traumatic event causing their disability or dysfunction. These reminders have a negative effect on the emotional state and the potential for immobility may bar the person from taking part in social activities. On the other hand, a mental disorder can have a negative effect on physical functioning leading to psychosomatic disease and a low functional outcome. We think that there is a close connection between the physical and psychological state of health with inevitable mutual interaction.

Both functional rehabilitation and psychosocial support play important roles while recovering from severe trauma. Several authors report positive correlations between low social support and the development or maintenance of PTSD [61–63]. These results are comparable to our findings with the HASPOC—a loss of the partner or friends as well as financial loss after the trauma occurs more often in the sample of patients with a diagnosed mental disorder.

Recruitment and retention are common problems in studies performed over a long-term period. As the study is based on volunteer participation, some kind of a pre-selection of only motivated probands must be taken into consideration. Concerning the subject of this study, several causes might be the reason for non-attendance (e.g. the probands do not want to be reminded of the accident or they are restrained to show their emotions). Probands of the older generation, especially, may have problems admitting and expressing their feelings. Another reason may be the fear of becoming stigmatised with a psychological diagnosis. The questionnaires are marked only with a number for allocation so that there is no direct link to the data of the probands. As a result, a lost questionnaire cannot be used by outsiders to draw any conclusions. Due to the long-term aspect of clinical studies, the possibility of blurred memories (recall bias) or extrusion mechanisms are increased and must be taken into consideration. Another limitation of our study is its successive design. Thus, we cannot provide information about the onset of the examined mental disorders. The only information we can provide is about the present status to the time of the succession study. Finally, we present single centre data that may be biased due to the therapeutic regimen of the institution.

The period of trauma, collecting the data and their interpretation is a potential drawback of this study. Since we report data of patients with treatment algorithms from 1973 to 1990 one might assume that there is a potential bias due to improved therapeutic strategies and concepts of today’s management. However, this is a large follow-up study investigating patients more than 10 years after injury and we thus will always be dealing with treatment algorithms at least on century or—more likely—2 centuries ago. One potential strategy would be a prospectively collected database of patients, e.g. 1, 2, 5 and 10 years after the injury. This would also be interesting in terms of a longitudinal investigation of the outcome.

These facts come also true for the latest PTSD treatment concepts since there have been some reasonable changes over the last decades.

Modern multimodal concepts consist of but are not limited to: treatment on ICU wards, out-patient follow-up and other concepts such as ICU diaries [64, 65]. In addition, there is evidence for trauma-focused cognitive-behavioural therapy in patients after critical illness or major trauma and ICU treatment [66]. However, only a limited fraction of patients are seeking psychological support due to various reasons including stigmatisation and embarrassment. Thus, internet-based interventions and protocols were developed over the past years with more than promising results. Internet-based strategies are able to overcome the trouble with stigmatisation and also availability of health care professionals which makes it a valuable option in patients suffering from PTSD [67]. Upcoming studies also deal with specifications such as internet-based cognitive-behavioural writing therapy [68].

Some authors and guidelines recommend a phase-based treatment for individuals—although there is promising literature, the phase-based strategy should not delay the onset of therapy [69].

Altogether, it seems more than likely that an early onset of the specific therapy after ICU treatment or even on the ICU ward is beneficial for the clinical course of the affected patients.

Despite its limitations, several strengths distinguish our work from previous research. For instance, it is characterised by its long-term period (a minimum of 14 years) and sample size (*n* = 324 probands). To our knowledge, long-term studies of this extent are sparse. Regarding the patient recruitment, we believe that a response rate of 53% is a respectable result and is appropriate for an outcome study with a minimum follow-up of 14 years. We cannot examine the effect of non-attendant patients on the outcomes of this study, as the attendant and non-attendant patients are comparable in demographic data and showed no considerable deviation. We, therefore, believe that the non-attendant patients do not bias the overall outcomes of this study, and the results obtained in this study are reliable.

## Conclusions

This study demonstrated that a remarkably high proportion of patients suffering from psychiatric sequelae could be expected, even after a follow-up of more than a decade following multiple traumas. As the study has shown, predictive factors can be different in long-term compared to short-term outcomes. The early detection of patients with a high risk for PTSD or MDD is of utmost importance for intervention and rehabilitation, which has the potential to prevent chronic mental disease.
